# What happened to financially sustainable firms in the Corona crisis?

**DOI:** 10.1007/s00550-020-00503-3

**Published:** 2020-07-22

**Authors:** Thomas Günther, Werner Gleißner, Christian Walkshäusl

**Affiliations:** 1 Technische Universität Dresden, Dresden, Germany; 2 FutureValue Group AG, Leinfelden-Echterdingen, Germany; 3 University of Regensburg, Regensburg, Germany

**Keywords:** Sustainability, Financial sustainability, Risk management, Risk governance, Earnings risk, Rating, D81, M41, M48

## Abstract

*Purpose: *Financial sustainability is underrepresented in both research on and the practice of sustainability management and reporting. In this article, we examine empirically how financially sustainable firms performed in the Corona crisis.

*Methods: *We measure financial sustainability by four conditions: (1) firm growth, (2) the company’s ability to survive, (3) an acceptable overall level of earnings risk exposure, and (4) an attractive earnings risk profile. We apply this measurement to investment portfolios of a broad sample of firms from 15 European countries of the MSCI Europe using typical investment portfolio characteristics.

*Results: *We find that financially sustainable firms outperform both the broad market and firms with low financial sustainability for the time span July 2019 to March 2020.

*Conclusion: *An investment strategy that invests in financially sustainable firms seems to be better capable of overcoming economic breakdowns such as the Corona crisis. We find that the returns increase with each of the four conditions that are included in the investment strategy. This underlines that considering financial sustainability is interesting for financial management, corporate governance and management control.

## Introduction: The Corona crisis and crisis resilience

The crisis caused by the Covid-19 virus has had tremendous impact on all major economies worldwide. Impact on US GDP for Q1 2020 is estimated to be −5.0%, with larger decline to be expected for Q2 2020. Since mid-March, more than 40 million people in the US have filed for unemployment. Similar impacts can be observed for all developed countries around the world. For some industries, such as airlines, hotels, and tourism, unprecedented declines in sales of more than 90% were seen, and thus heavy losses concerning EBIT and net earnings emerged. The stock market plummeted by around 40% within weeks. Some firms like Lufthansa, Adidas, or US airlines had to look for financial support by the government to be capable of surviving. However, there are other firms profiting from the crisis, as for example E‑business firms such as Amazon, Netflix, and Zoom as well as pharmaceutical or medical companies such as Gilead Sciences, AbbVie, and Draegerwerk.

Is this just by chance when a firm is in the right industry, or do we find systematic reasons behind “poor” or “good” resilience in crises such as the Corona crisis? In a recent paper we suggested four conditions for *financial sustainability* as one dimension of the widely accepted concept of *sustainability.* However, sustainability is mostly used in general to address ecological and social issues, and to date financial sustainability has been the focus of neither research nor practice.

This study analyses what happened to firms that had been classified as “financially sustainable” before the Corona crisis.

## Financial sustainability and crisis resilience

*Sustainability* has been widely discussed in the academic literature and in practice, and sustainability has become a widely accepted buzzword in both companies and society at large. Meanwhile, *sustainability* is deeply grounded as a top management objective in corporate governance. For example, since the version dated June 18, 2009, the German Corporate Governance Codex (GCGC) has been changed to express, besides the commitment of the management to the company and its shareholders, an explicit commitment to other stakeholders of the firm: the management board “considers the needs of the shareholders, the employees and other stakeholders, with the objective of sustainable value creation” (German Corporate Governance Code, Sect. 4.1.1.). The explicit shareholder orientation of the original GCGC is replaced by a much broader stakeholder orientation, while at the same time changing the primary objective of the firm from generating a “sustainable company value” to the rather vague objective of “sustainable value creation”. The GCGC strictly only applies to German corporations listed on the stock exchange and to other capital market-oriented companies (e.g., firms with bonds on the capital market, but no shares on the stock exchange). Nevertheless, the GCGC stands for “good corporate governance” of firms regardless of their legal entity, and thus it has a signalling function for other legal forms of firms. According to Sect. 161 (1) of the German Stock Corporation Act (AktG), capital market-oriented companies must explain in an additional statement whether they comply with the recommendations of the GCGC (so-called “comply or explain” rule) or justify any deviations from the GCGC. This makes the GCGC effectively legally binding.

In methodological terms, the GCGC raises the question of how “sustainable value creation”, the primary objective of the firm following the GCGC, can be measured in practice. The GCGC itself is rather silent on the actual operationalization. Günther, Endrikat, and Günther (2016) differentiate between a time and a scope dimension when disentangling sustainability for management and management control use. The time dimension differentiates between intergenerational and intragenerational justice. *Intergenerational justice* is derived from the so-called Brundtland Commission of the United Nations, which states that “sustainable development” must “ensure that it meets the needs of the present [generation] without compromising the ability of future generations to meet their own needs” (Brundtland Commission 1987, p. 41). *Intragenerational justice* is based on the requirement that within any (but especially within one distinctive) generation, economic activities must be such that both organisations and individuals adequately address all three dimensions of the so-called *triple bottom line* of social, ecological, and economic (or financial) sustainability and, thus, create intragenerational justice. The triple bottom line approach constitutes the scope dimension of sustainability (see the basic definition in Carroll 1979, p. 500).

These three sustainability goals are also addressed as the three pillars of sustainability or the three Ps: people, planet, and profit. In addition, *corporate social responsibility* (CSR) or *corporate social performance* (CSP) are used as umbrella terms capturing the simultaneous management of all three pillars of sustainability. Furthermore, the *ESG* concept (ecological, social, governance) focuses only on environment and social issues but not economic ones; however, it adds the governance perspective. However, economic (or financial) sustainability is often scarcely addressed in both sustainability management and sustainability reporting in practice, as well as in academic sustainability research, if it is considered at all. Therefore, it makes sense to close this gap for research and practice (Gleißner et al. 2020).

In addition to sustainability management, an operationalization for financial sustainability is also supportive for risk management (Lenssen et al. 2014), especially for the strategic and holistically oriented risk-governance approach (e.g., Stein and Wiedemann 2016; Hiebl 2019). So far, risk management has been mainly addressed in terms of its contribution to company value (e.g., Grace et al. 2015; McShane et al. 2011). However, company value records the objectives of risk management incompletely because it does not incorporate risk-limitation goals. This incompleteness can be remedied by the measurement of financial sustainability as presented by Gleissner et al. (2020), which is the conceptual foundation of this article. Financial sustainability as measured below becomes a control parameter for both risk and sustainability management, and it is capable of adequately expressing the benefits of risk-governance concepts, as validated in a long-term empirical study by Gleissner et al. (2020).

## Conditions of financial sustainability and assumptions of the model

The main focus of this study is to analyse how financially sustainable firms survived the Corona crisis. To do so, financial sustainability as one dimension of the triple bottom line has to be measured and operationalized.

When the basic idea of the Brundtland Commission on sustainable development is applied to financial sustainability as one dimension of the triple bottom line, *financial sustainability* means that companies must be financially managed such that present financial success is ensured without jeopardising financial success in the future, including success for future generations (see Günther and Günther 2017, p. 5). Thus, the goal of financial sustainability is similar to the goal of long-term, future-oriented value creation (or better, value preservation).

Financial sustainability can also be seen as a measure of risk management. Securing the going concern of the company, and thus ensuring financial sustainability, can be seen as the primary goal of risk management. Thus, measuring financial sustainability is relevant to risk management because it allows for operationalising its primary goal. Based on Sect. 91 of the German Stock Corporation Act (AktG), companies must recognise any “developments that put the continued existence of the company at risk” at an early stage and establish a risk-management system, securing the going concern of the company as a core aspect of financial sustainability. Gleißner et al. (2020) show that financial sustainability has to go beyond securing the continued existence of the company by adding additional criteria, as for example the sustainable attractiveness of the company for its owners. Thus, besides the primary objective of the firm to maximise its company value, financial sustainability takes over the secondary boundary condition of ensuring the long-term financial survival of the firm.

The measurement of financial sustainability is of special importance for the so-called business judgement rule, for Germany codified in Sect. 93 of the Stock Corporation Act (AktG). Changes to the risk exposure resulting from a “corporate decision” must be identified and quantitatively assessed prior to that decision as part of “good financial management”. If a decision endangers the future going concern of the company, it is crucial for the management to point out the consequences of such a decision (Graumann et al. 2009; Gleißner 2019).

Based on these assumptions and the general understanding of financial sustainability as financial management of the firm such that present financial success is ensured without jeopardising financial success in the future, including success for future generations, the following four requirements for financial sustainability can be derived:

### Real preservation of the company (growth > inflation rate)

From an understanding of financial sustainability as long-term survival of the firm, it follows that the company does not disappear “as planned”. Such a disappearance is to be expected when a negative growth rate is anticipated. Not only a negative nominal growth but actually a negative real growth is problematic in this case. In other words, a company may be called financially sustainable only if the company permanently achieves a growth rate of at least the level of expected inflation (e.g., in the continuation phase of company valuation).

In detail, we measure the net income growth rate *g* over the prior five years and require this growth rate *g* to be greater than the target inflation rate of 2% as a proxy for a five-year average inflation rate. The net incomes used for the calculation must be positive.

$$\mathrm{g}=\left(\frac{NI_{t}}{NI_{t-5}}\right)^{\frac{1}{5}}-1\geq 0,02$$

### A company’s ability to survive without making demands on its owners (sufficient survival probability of the company)

Financial sustainability requires the long-term survival of the company, which is the core task of risk management. The implementation of a risk-management system was introduced for Germany in 1998 by the Corporate Sector Supervision and Transparency Act (KonTraG) and the IDW (Institute of Public Auditors in Germany) audit standard for the early risk-identification system (IDW PS 340), in accordance with Sect. 317 (4) of the German Commercial Code (HGB) (Hannemann et al. 2013; Gleißner 2018). Sect. 91 (2) of the Stock Corporation Act (AktG) requires that “the Board must take appropriate actions … in particular establish a monitoring system, so the developments that pose a threat to the continued existence of the company are detected early on”. The identification, quantification, management, and monitoring of material firm risks, as well as risk aggregation to determine the overall risk exposure of the company, are regarded as essential tasks of a risk-management system, especially of an early-risk-detection system. Risk aggregation can be considered a crucial task of these early-risk-detection systems, as developments that threaten a company’s going concern mostly result from the combined effects of individual risks, and a company’s ability to survive therefore depends on the aggregate risk exposure (Angermüller et al. 2020).

Hence, financial sustainability can be operationalised by the probability of a company’s survival and the avoidance of “developments that put the existence of the company at risk” (in the sense of Sect. 91 (2) of the AktG). Thus, methods of insolvency forecasting can be applied. Therefore, as a second condition for financial sustainability, the insolvency probability of a firm (probability of default) has to be measured, for example expressed by a company rating. The scholarly literature on company valuation speaks of an insolvency risk or distress risk in this context (Friedrich 2015; Lahmann et al. 2018).

For this study, the probability of insolvency *p*, as the inverse of the probability of survival, is calculated using a logistical function based on previous research findings (Blum et al. 2005; Bemmann 2007), consisting of two indicators, the equity ratio (ER) and the total return on capital employed (ROCE). The probability of insolvency *p* is supposed to be below 1%, which corresponds to a BB rating or better (following the Standard & Poor’s rating table), deduced from an understanding that financially sustainable companies should have above-average ratings. Alternatively, other models for insolvency forecasting can be used (e.g., see the models of Altman 2000; or Ohlson 1980).

$$p=\frac{0.265}{1+e^{-0.41+7.42(ER)+11.2(\mathrm{ROCE})}}\leq 0.01$$

### Total earnings risk exposure acceptable to owners

The consideration of the probability of survival alone is not sufficient for the assessment of a company’s total risk exposure from the perspective of its owners, as the probability of default captures only the worst case of a downside risk. Risk-averse owners will prefer less risky investment opportunities to riskier ones. Thus, it is necessary to consider the company’s earnings risk that can be expressed by measures of risk such as the standard deviation, the variation coefficient, or the value-at-risk of the cash flows. Therefore, a company can be regarded as financially sustainable if its earnings risk exposure is acceptable to its owners. Thus, the questions arise: what risk measure can be used to express the risk preferences of the owners (e.g., Renn 2008; Slovic 1987; Sarin and Weber 1993), and what level of risk exposure (measured by aggregating all risks of a firm) are owners willing to take?

Limitations for acceptable risk exposure are discussed in the safety-first approaches of financial management (e.g., Roy 1952; Telser 1955; Campbell et al. 2008) and in so-called risk-tolerance concepts developed by the interpretation of legal requirements (e.g., Gleißner and Wolfrum 2017) of IDW PS 981, also integrated into the DIIR Revision Standards No. 2. For the operationalisation of this third condition, different approaches can be used, such as risk of loss (e.g., value-at-risk for earnings, which is covered by a minimum equity capital requirement to buffer potential losses and avoid over-indebtedness according to insolvency law, for example InsO for Germany) or earnings volatility (i.e., potential deviations from planned annual net earnings).

For this study, we chose an earnings volatility measure as the variation coefficient of net earnings measured as the standard deviation of net income *σ(NI)*, measured over a five-year period, scaled by its mean *(NI*_*t,t*_ _*−*_ _*5*_*)* over the same period. The measure must be positive and below the threshold of 40% for a firm to be classified as having a low earnings risk and, thus, as being financially sustainable.

$$C=\frac{\sigma \left(NI\right)}{\overline{NI_{t,t-5}}}\leq 0,4$$

### Economic interest of the owners in a lasting continuation of the company (attractive return–risk profile)

A fourth aspect of financial sustainability is the interest of owners in maintaining their company on a long-term basis. An acceptable risk exposure itself is not sufficient, as the accepted risks should be covered by adequate earnings. Shareholders will be interested in sustaining their investment in the firm when the investment is economically reasonable. This can be regarded as given when the company offers an attractive return–risk profile to its owners, in comparison with alternative investment opportunities. Only an economically attractive company will be operated on a long-term basis. The alternative is the liquidation of the firm.

In a real, imperfect capital market, it is reasonable to compare expected returns with the risk-adjusted cost of capital required for that return. The cost of capital should reflect the actual aggregated earnings risks of the firm and not historical stock return fluctuations—as for example reflected by the CAPM approach. Shareholders will continue with the company when its expected returns are higher than the expected return of alternative investment opportunities with the same risk. In other words, the expected average return must be above the risk-adjusted cost of capital. In a multi-period context, this results in demand for a fundamental capitalised earnings value greater than the net asset value (reproduction or liquidation value) of the equity capital. As a proxy, instead of the net asset value the book value of equity can be used.

For this study, we define the fourth condition of an attractive return–risk profile when the fundamental earnings value V is above the book value of equity (“*Equity*”), where *i* represents the risk-adjusted cost of capital and *p* the probability of insolvency (for further details see Gleißner et al. 2020; and Dorfleitner and Gleißner 2018).

$$V=\frac{NI_{t,t-5}\left(1-p\right)}{i+p}\geq \textit{Equity}$$

with

$$i=\frac{1+r_{f}}{1-\lambda \cdot \frac{\sigma \left(Z\right)}{E\left(Z\right)}\cdot d}-1=\frac{1+r_{f}}{1-\lambda \cdot C\cdot d}-1\approx r_{f}+\lambda \cdot C\cdot d.$$

For the empirical study, a sustainable net income NI_t,t_ _−_ _5_ is discounted, which is determined as the mean net income over the last five years. Given the probability of default *p* and the cost of capital *i*, both assumed to be constant, the fundamental earnings value V can be calculated (see Gleißner et al. 2020). The potential added value of company growth is neglected here. Furthermore, a sustainable net income and the net present value neutrality of future net investments are assumed.

The parameters for the calculation of the cost of capital *i* are defined as follows. First, the rate of return on the risk-free investment *r*_*f*_ is equal to 3% per year, which approximately corresponds to the long-term average of the one-month Fibor/Euribor rates over the 1990–2019 sample period of the underlying longitudinal study of Gleißner et al. (2020). Second, motivated by the extensive evidence in Dimson et al. (2002), the parameter of the risk–return profile λ is set to 0.25. The parameter *λ* expresses the risk–return profile of alternative investments and shows the additional return per unit risk in alternative investments (the so-called Sharpe ratio, see Gleißner et al. 2020). Lambda equals the ratio of the market risk premium (r_M_ − r_F_) to the standard deviation of r_M_. Lambda λ = 0.25 means that an increase in the volatility C by one percentage point is accompanied by an increase in the expected excess returns by one-fourth of a percentage point. Third, we assume a diversification factor of *d* = 0.5 (based on results by Gleißner and Walkshäusl 2018).

To sum up, financial sustainability can be regarded as a complex latent construct—that is, a construct which cannot be directly observed but which can be indirectly measured by various indicators such as the four proposed conditions. The understanding of the term “financial sustainability”, based on the earlier conceptualization of Günther and Günther (2017), can therefore be summarised as follows: a financially sustainable company has a high probability of survival (low probability of insolvency), a relatively low earnings risk, a positive real growth rate, and a risk–return profile that makes it sufficiently attractive for its owners.

## Results for the empirical evidence for the behaviour of financially sustainable firms in the Corona crisis

Based on the study assessing the long-term effects of firms concerning financial sustainability (see in detail Gleißner et al. 2020), first we examine whether the sample firms fulfil the four conditions as defined above, ending June 2019. The sample covers all listed stocks of 15 industrial European countries, in line with the MSCI Europe classification. Monthly returns, including reinvested dividends, are considered according to Thomson Reuters Datastream for the period July 2019 to March 2020. The annual financial accounting data employed are from Wordscope. All firms included have a positive book value of equity. Financial companies (i.e., banks or insurance firms) are excluded from the analysis.

At the end of June 2019, companies are assessed for their financial sustainability as measured by the four proposed conditions based on the available financial accounting data of the previous fiscal year. In detail, we use equity ratio (ER), return on capital employed (ROCE), and net income after tax in year *t* (*NI*_*t*_) to operationalise the four conditions as described above. If one condition is met, a company receives one point in the corresponding category. Thus, a Score 0 firm is a firm which does not fulfil any of the four conditions, whereas a Score 3 firm is a firm fulfilling three out of the four conditions.

**Fig. 1:**
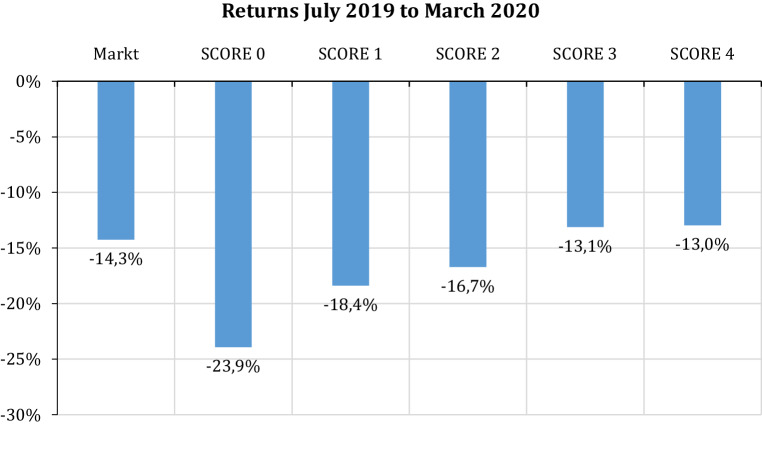
Returns of market and Score 0 to Score 4 portfolios, July 2019 to March 2020

Fig. 1 shows that Score 0 firms, that are firms that do not fulfil any of the four conditions of financial sustainability, perform worst in the Corona crisis, resulting in a return of −23.9% for the period July 2019 to March 2020. In contrast, Score 4 firms perform best, with an average return of −13.0%. All Score portfolios have losses, but also the market realizes average losses of −14.3%. Thus, Score 0 firms perform worse than the market average and Score 4 firms perform better. Fig. 1 also shows that the average loss decreased with increasing fulfilment of the four conditions for financial sustainability (from Score 0 to Score 4). We conclude that financial sustainability protects firms against the crisis and creates financial resilience.

**Fig. 2:**
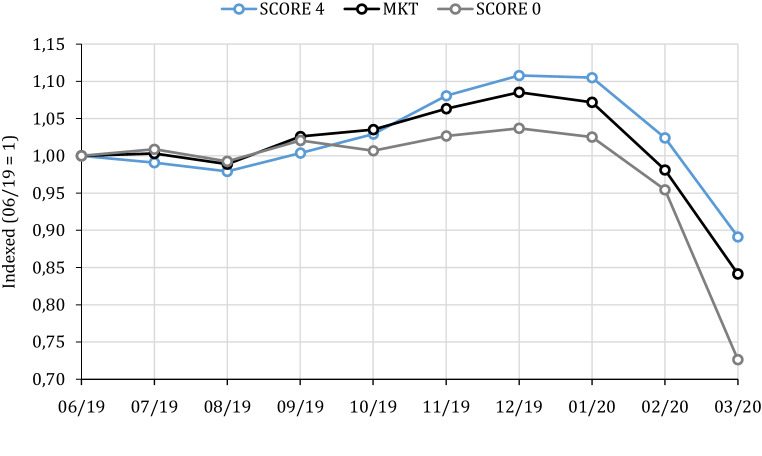
Development of indexed portfolios, July 2019 to March 2020

Fig. 2 shows the development over this nine-month period. Score 4 firms perform better than the market average even before the outbreak of the Corona crisis, whereas Score 0 firms perform worse. In the crisis all firms lose, but Score 4 firms lose less than the market and less than Score 0 firms. Again, financial sustainability seems to protect firms in the crisis and lower losses for owners.

Table 1 shows the return–risk characteristics of the European market (MSCI Europe), of the five Score portfolios (Score 0 to Score 4), and the differences of the portfolios, Score 4 to Score 0 (Diff), for the period 07/2019–03/2020 in which the Corona crisis unfolded. We deliver, each on a monthly basis, the arithmetic mean returns (µ), the volatility of the returns ($\sigma$), and the *p* value for the null hypothesis, that the mean return equals zero as well as the geometric mean return ($\overline{r}_{G}$). Table 1 shows that the average returns per month (µ or $\overline{r}_{G}$) are increasing with increasing fulfilment of financial sustainability conditions (i.e., from Score 0 to Score 4). In contrast, the volatility of returns ($\sigma$) is shrinking.

**Table 1:** Return–risk characteristics of Score × portfolios, 07/2019–03/2020

	Market	Score 0	Score 1	Score 2	Score 3	Score 4	Diff
µ	−1.74	−3.13	−2.13	−2.02	−1.62	−1.12	2.01
$\sigma$	5.89	8.30	6.99	6.72	5.54	5.67	3.79
$p-\text{Wert}(\upmu =0)$	0.40	0.29	0.39	0.39	0.41	0.57	0.15
$\overline{r}_{G}$	−1.90	−3.49	−2.37	−2.24	−1.76	−1.27	1.96
*Characteristics:*							
Observations	–	265	464	426	460	405	–
Size	–	1624	2200	2639	4448	3796	–
B/M	–	0.70	0.73	0.55	0.44	0.35	–
MOM	–	−0.03	0.08	0.09	0.05	0.07	–
ROE	–	−0.10	0.12	0.17	0.22	0.21	–
*CAPM regression analysis:*							
$\alpha$	–	−0.82	−0.11	−0.05	0.00	0.51	1.33
$p-\text{Wert}(\alpha =0)$	–	0.43	0.83	0.83	0.99	0.28	0.27
$\beta$	–	1.34	1.17	1.13	0.93	0.94	−0.40
$p-\text{Wert}(\beta =1)$	–	0.09	0.08	0.02	0.15	0.46	0.00
$\mathrm{Adj}. R^{2}$	–	0.89	0.96	0.99	0.99	0.95	0.29

The characteristics show for each Score portfolio the number of observations, the average size of the firms measured by market capitalization (in Mill. EUR), the book-to-market ratio (B/M), the momentum (MOM) on the basis of the performance over the last 12 months, and the profitability of the firm based on return on equity (ROE), ending June 2019. Table 1 shows that the number of observations for Score 1 to Score 4 shows similar group sizes, whereas the group of Score 0 firms is smaller. B/M ratios are declining and ROEs are increasing from Score 0 to Score 4 firms.

For the CAPM regression analysis, the returns of the Score portfolios are regressed on the market returns. The risk-free return rate is assessed with zero. Alpha α is the risk-adjusted return per month, and the related *p*-value tests the null hypothesis, that α equals zero. Interestingly, abnormal returns are again increasing with the scores on financial sustainability. Beta β captures the sensitivity of the depending returns relative to the market portfolio, and the related *p*-value tests the null hypothesis that β equals one. Score 4 firms have lower Beta values than Score 0 firms. The adjusted *R*^*2*^ shows significant explanatory power of the analysed models.

## Conclusions for financial management, corporate governance, and management control

In general, the results show that financial sustainability is not only a long-term indicator for “good” financial management, as shown by Gleißner et al. (2020), but also a suitable indicator for financial resilience in crises situations such as the Corona crisis. Financial sustainability takes over the role of “blood” tests for the financial health of a firm.

In detail, the following conclusions can be drawn:

- Financial sustainability measured by the four suggested conditions is a valid indicator for long-term but also for short-term financial stability and resilience of a firm. Firms which fulfilled the conditions of financial sustainability before the crisis also perform significantly better during the crisis.
- The four conditions of financial sustainability are in line with classical principles of financial management such as rating assessments or insolvency forecasting, determination of the total risk exposure of the firm, and an adequate fit of the earnings and risk potential of a firm.
- The defining conditions of financial sustainability are in accordance with legal requirements of corporate governance and especially of risk governance, such as the demand for an adequate risk management system (e.g., Sect. 91 of the AktG), the business judgement rule (e.g., Sect. 93 of the AktG), or the undefined objective of the German Corporate Governance Codex to “sustainable value creation” for all stakeholders of the firm.
- In our study, the four conditions were applied to the past performance of the firms by using archival data. The four conditions can also be applied to corporate planning to assess the near-term financial sustainability of the firm.
- The suggested operationalisation can be modified and expanded, for example by using Monte Carlo simulation approaches, to better calibrate the probability of insolvency, the risk exposure, or the return–risk profile of the firm.
- The underlying drivers of the four conditions of financial sustainability can be used to better control the financial situation of the firm, for example, by analysing the impact of corporate decisions (e.g., investments or M&As) on the probability of default, the risk exposure, or the return–risk profile of the firm, and, thus, finally on financial sustainability.
